# Calcium oscillations in HEK293 cells lacking SOCE suggest the existence of a balanced regulation of IP_3_ production and degradation

**DOI:** 10.3389/fsysb.2024.1343006

**Published:** 2024-03-15

**Authors:** Clara Octors, Ryan E. Yoast, Scott M. Emrich, Mohamed Trebak, James Sneyd

**Affiliations:** ^1^ Department of Mathematical Biology and Physical Chemistry, Science Faculty, Université Libre de Bruxelles (ULB), Brussels, Belgium; ^2^ Graduate Program in Cellular and Molecular Physiology, Pennsylvania State University College of Medicine, Hershey, PA, United States; ^3^ Department of Pharmacology and Chemical Biology, University of Pittsburgh School of Medicine, Pittsburgh, PA, United States; ^4^ Vascular Medicine Institute, University of Pittsburgh School of Medicine, Pittsburgh, PA, United States; ^5^ UPMC Hillman Cancer Center, University of Pittsburgh School of Medicine, Pittsburgh, PA, United States; ^6^ Department of Mathematics, University of Auckland, Auckland, New Zealand

**Keywords:** calcium oscillation, calcium influx, HEK293 cells, SOCE, PLC regulation, IP3, STIM, bifurcation analysis

## Abstract

The concentration of free cytosolic Ca^2+^ is a critical second messenger in almost every cell type, with the signal often being carried by the period of oscillations, or spikes, in the cytosolic Ca^2+^ concentration. We have previously studied how Ca^2+^ influx across the plasma membrane affects the period and shape of Ca^2+^ oscillations in HEK293 cells. However, our theoretical work was unable to explain how the shape of Ca^2+^ oscillations could change qualitatively, from thin spikes to broad oscillations, during the course of a single time series. Such qualitative changes in oscillation shape are a common feature of HEK293 cells in which STIM1 and 2 have been knocked out. Here, we present an extended version of our earlier model that suggests that such time-dependent qualitative changes in oscillation shape might be the result of balanced positive and negative feedback from Ca^2+^ to the production and degradation of inositol trisphosphate.

## 1 Introduction

Variations in the intracellular concentration of calcium ([Ca^2+^]) have been identified as an important control mechanism in various cell types, and regulate a variety of essential cellular functions (Berridge et al., 2003). These cytosolic fluctuations of [Ca^2+^] can take multiple shapes, including oscillations and periodic spikes, and arise from Ca^2+^ transport into and out of the endoplasmic reticulum (ER) and across the plasma membrane (PM) (Dupont et al., 2016). Their frequency and shape is modulated by a host of factors, including oscillatory Ca^2+^ influx and mitochondrial transport (Yoast et al., 2021; Benson et al., 2023).

Calcium influx (*J*_in_) is a crucial mechanism for control of a plethora of physiological functions. Not only is Ca^2+^ influx necessary for long-term Ca^2+^ oscillations, it also has significant effects on oscillation shape and frequency (Girard and Clapham, 1993; Yao and Parker, 1994). The most ubiquitous pathway for Ca^2+^ entry in non-excitable cells is store-operated Ca^2+^ entry, or SOCE (Lewis, 2007; Smyth et al., 2010; Prakriya and Lewis, 2015). As Ca^2+^ in the ER is depleted, stromal interaction molecules (proteins existing in two isoforms in mammals, STIM1 and 2) on the ER membrane aggregate in regions of the ER membrane close to the PM. By interacting with Orai proteins on the PM (existing in three isoforms in mammals, Orai1, 2, and 3), they form a pore channel allowing extracellular calcium to enter the cytosol thus mediating the so-called Ca^2+^-release-activated current, *I*_crac_ (Lewis and Cahalan, 1989; Hoth and Penner, 1992).

To clarify more precisely the functions of the different Orai isoforms, Yoast et al. (2020) genetically modified wild-type HEK293 cells using CRISPR/Cas9 biotechnology and generated clonal cells missing one, two or three Orai isoforms. They then performed single and increasing-dose response experiments on both the knock-outs and wild-type cells to study how each isoform contributes to, and regulates, Ca^2+^ oscillations.

To help understand these experimental data, Yoast et al. (2020) also constructed a mathematical model based on the model in Sneyd et al. (2017). Despite generally good agreement between modeling and experimental results, certain behaviors could not be reproduced by the model of Yoast et al. (2020). In particular, 10% of the cells where Orai1 was knocked out exhibited broad spikes that are characterized by a prolonged decrease in cytosolic calcium, but such behavior could not be explained by the model. Following a similar experimental procedure as in Yoast et al. (2020), Emrich et al. (2021) knocked out the STIM1 and/or 2 isoforms to gain better insight into the regulation of SOCE (Emrich et al., 2021). They developed a model which is in good agreement with the main features of the experimental results, although which is unable to reproduce the full range of observed responses. In particular, HEK293 cells lacking STIM1 and 2 but having intact Orai isoforms (i.e., STIM1/2-KO cells) exhibit behaviors that could not be reproduced by the model proposed in Emrich et al. (2021). This includes, for example, the coexistence of qualitatively different oscillations in a single time series.

Here, we extend the model of Emrich et al. (2021) to get a better understanding of the possible mechanisms underlying the responses seen in STIM1/2-KO cells. Firstly, we will define a new classification for the data collected in Emrich et al. (2021). Then, a modified version of the model will be proposed to explain the experimental data. Finally, we provide a qualitative dynamical analysis of the model.

## 2 Data

**FIGURE 1:**
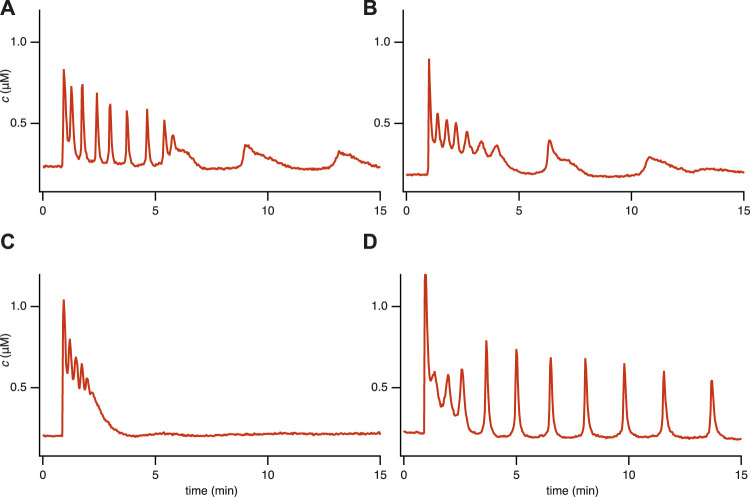
Experimental time series for 4 representative [Ca^2+^] responses in STIM1/2-KO HEK293 cells after stimulation by 10 *μ*M of carbachol (CCh) at 1 min in a medium containing 2 mM Ca^2+^. We characterize these different responses as **(A)** Fast spike oscillations followed by broad spikes, **(B)** Oscillation on a raised baseline followed by broad spikes, **(C)** No steady-state response, **(D)** Oscillations. These categories are not well defined, and it is thus not possible to characterise all the cell responses in a precise and unambiguous manner. Nevertheless, these four categories are useful. Data from Emrich et al. (2021).

**FIGURE 2:**
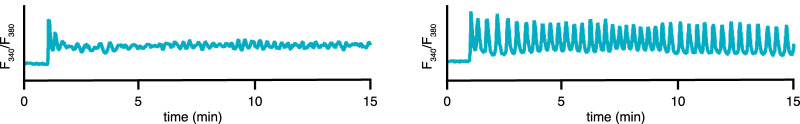
Experimental time series for 2 representative cells/conditions of the cytosolic [Ca^2+^] in STIM1/2-KO HEK293 cells after stimulation by 10 *μ*M of CCh at 1 min. Medium containing 2 mM Ca^2+^ and 1 mM of gadolinium (known to close the cell at this concentration (Yoast et al., 2020)). **(A)** a plateau response, **(B)** a fast oscillation on a raised baseline. Slower oscillations, not on a raised baseline, are also observed (data not shown). Data from Emrich et al. (2021).

Emrich et al. (2021) studied the cytosolic calcium responses of STIM1/2-KO HEK293 cells in both the presence (open-cell, Figure 1) and absence (closed-cell, Figure 2) of Ca^2+^ fluxes across the cell membrane. While certain aspects of the responses were highlighted, others remained unexplored in their investigation.

One behavior that Emrich et al. considered is the increased frequency of oscillations in STIM1/2 KO cells (Emrich et al. (2021); Figure 1, panel G) compared to the STIM1 KO or STIM 2 KO cells at an equivalent stimulation level (Emrich et al. (2021); Figure 1, panels D, E, F). The investigation of this unexpected behavior ultimately revealed that STIM1/2-KO cells are more sensitive to IP_3_-mediated Ca^2+^ release. Furthermore, the study demonstrated that unactivated STIM1 and unactivated STIM2 both inhibit IP_3_R-mediated Ca^2+^ release, thus contributing to the fast oscillations in STIM1/2-KO cells.

However, we performed a new analysis of the single-dose experimental data that revealed behaviors in STIM1/2-KO cells that could not be reproduced by the model in Emrich et al. (2021). Based on a qualitative classification, we grouped all the responses into four representative categories (see Figure 1 and refer to Supplementary Appendix S6.1; Table 1 for detailed information). Our observations indicate the coexistence of qualitatively different oscillations within a single time series, including the emergence of broad spikes following fast spike oscillations or oscillations on a raised baseline (Figures 1A,B). This observation brings an additional layer of complexity to the cellular dynamics that was not previously addressed in Emrich et al. (2021). Our study aims to complete this description by characterizing these behaviors using our newly developed model.

**TABLE 1:** Classification of the Ca^2+^ responses for 2 sets of 120 STIM1/2-KO cells after stimulation by 10 *μ* M of CCh during a single-dose experiment in open-cell conditions. Columns A, B, C, and D refer to the representative behavior illustrated in Figure 1A–D, respectively.

	A	B	C	D
Set 1	28	33	45	14
	A	B	C	D
Set 2	22	30	39	29

## 3 Model formulation

To study the responses of STIM1/2-KO cells, we develop a model based on the previous work of Sneyd et al. (2017), Emrich et al. (2021) and Yoast et al. (2020). In our model, agonist stimulation activates phospholipase C (PLC) which produces inositol trisphosphate (IP_3_), thus releasing Ca^2+^ from the ER. Calcium is removed from the cytoplasm by SERCA pumps in the ER membrane, and PMCA pumps in the plasma membrane. Oscillations in [Ca^2+^] arise, firstly, from the activation and inactivation of the IP_3_ receptor by Ca^2+^, and, secondly, from feedback between [Ca^2+^] and the production and degradation of IP_3_. Both these mechanisms are well known and have been previously modeled in detail in multiple places (Dupont et al., 2016).

Where our model differs from previous modeling work is our fundamental assumption that, for a fixed agonist stimulation, the steady-state [IP_3_] depends on [Ca^2+^] in a biphasic manner. When [Ca^2+^] is low an increase in [Ca^2+^] will increase the steady-state [IP_3_], while when [Ca^2+^] is high an increase in [Ca^2+^] will decrease the steady-state [IP_3_]. Previous modeling work has allowed for such a possibility, but has not (to our knowledge) explored in depth the consequences of such an assumption. As we show here, it is this assumption that allows for Ca^2+^ oscillations that change in character over a single time course.

We incorporate this assumption in our model by including Ca^2+^-dependence of both the production and the degradation of IP_3_. It has been demonstrated that an increase in cytosolic calcium can lead to a significant rise in PLC activity (Horowitz et al., 2005), resulting in an augmentation of IP_3_ production. Conversely, IP_3_ degradation can occur, notably through the action of a 3-kinase that is itself regulated by calcium (Berridge and Irvine, 1989). Full details of the model equations are given in Supplementary Appendix S6.2.

## 4 Results

**FIGURE 3:**
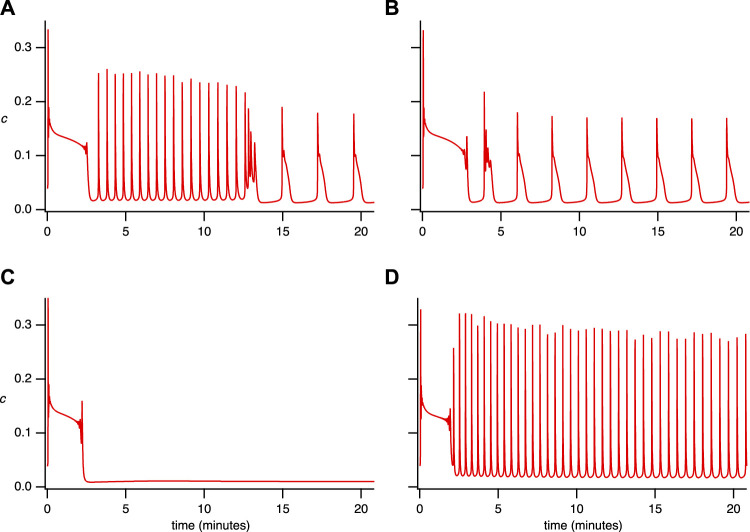
Simulated time series for the single-dose experiment. The parameter set used is in Supplementary Appendix S6.2, Table 2, with **(A)** *V*_PLC_ = 0.0145 *μ*M s^−1^. **(B)** *V*_PLC_ = 0.015 *μ*M s^−1^. **(C)** *V*_PLC_ = 0.014 *μ*M s^−1^, *α*_0_ = 0.0006 *μ*M s^−1^, *V*_PM_ =0.12 *μ*M s^−1^, *K*_PM_ = 0.14 *μ*M. **(D)** *V*_PLC_ = 0.014 *μ*M s^−1^.

Using the parameter set from Table 2 (Supplementary Appendix S6.2), we simulated single-dose responses with the software XPPAUT (Figure 3) (Ermentrout, 2002).

**TABLE 2:** Model parameters. The values in braces are used to generate the plots in Figure 3C. All concentrations are in *μ*M and time is in seconds. These parameters are taken from previous models (Sneyd et al., 2017; Cloete et al., 2021; Emrich et al., 2021), slightly adjusted to obtain qualitatively accurate oscillation periods.

Parameter	Value	Parameter	Value
*α*_0_	0.007 (0.0006)	*δ*	0.176
*V*_PM_	0.186 (0.12)	*K*_PM_	0.2 (0.14)
*V*_SERCA_	0.36	*K*_SERCA_	0.2
*K* ^^^	0.00001957	*τ*_max_	75
*k*_*f*_	1.2	*γ*	55
*K*_*c*_	0.14	*K*_*h*_	0.08
*K*_*τ*_	0.1	*K*_*n*_	0.214
*V*_PLC_	0.014	*K*_*p*_	0.2 *μ*M
*K*_*g*_	0.275	*A*_2_	0.104

Figure 3 illustrates that the model can reproduce (qualitatively) the primary features observed in the experimental data from the single-dose experiment (Figure 1). Notably, it accurately captures the coexistence of various types of oscillations (Figures 3A,B). Moreover, it reproduces the observed diversity of cellular behavior, such as the emergence of broad spikes (Figures 3A,B), the absence of steady-state response (Figure 3C), and the narrow spike oscillation (Figure 3D). These findings demonstrate the model’s ability to replicate aspects that the model in Emrich et al. (2021) could not.

Since, we can reproduce a variety of behaviours by varying a single parameter, *V*_PLC_, our simulations thus predict that the diversity of cellular behavior observed in Figure 1 may be explained in large part by intercellular variations in PLC activity (although variations in other parameters are not ruled out, of course). Despite the absence of SOCE and minimal calcium influx (*α*_0_ = 0.007), the model demonstrates the existence of maintained broad and narrow spike oscillations, mirroring experimental data. Setting *α*_0_ = 0 in simulations eliminates all forms of sustained oscillations (results not shown), thus emphasising the importance of Ca^2+^ influx for the maintenance of long-term oscillations.

**FIGURE 4:**
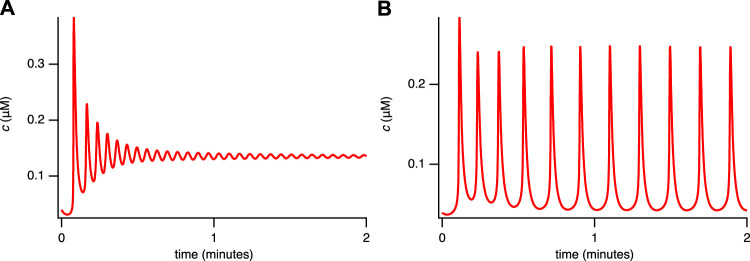
Solutions of the closed-cell model for the single-dose experiment. **(A)** *c*_*t*_ = 1.5 *μ*M, *V*_PLC_ = 0.005 *μ*M s^−1^. **(B)** *c*_*t*_ = 1 *μ*M, *V*_PLC_ = 0.013 *μ*M s^−1^. The other parameters are given in Supplementary Appendix S6.2, Table 2.

A closed-cell version of the model can be constructed by setting *δ* = 0 so that $\frac{dc_{t}}{dt}=0$ , i.e., the total calcium in the cell remains constant. Physiologically, this corresponds to abrogating the fluxes across the plasma membrane (*J*_in_ = *J*_pm_ = 0). The closed-cell version of the model successfully reproduces the main features observed in the insulation (closed cell) experiment (Figure 2), namely, plateaus (Figure 4A) and rapid oscillations (Figure 4B). The experimental findings indicated that closing the cell abolishes broad-spike oscillations while preserving narrow spikes and enabling the emergence of plateaus (Emrich et al., 2021).

### 4.1 Dynamical structure of the model

In order to understand better the dynamical structure of our model, and to relate this to physiological behavior, we use a multiple-time-scale approach that relies on the fact that Ca^2+^ transport across the plasma membrane is often significantly slower than Ca^2+^ transport across the ER membrane. Indeed, in many cell types Ca^2+^ oscillations continue for many periods in the absence of Ca^2+^ influx, thus demonstrating that the plasma membrane Ca^2+^ pumps work more slowly than do the SERCA pumps. In this approach, *c*_*t*_ is treated as a bifurcation parameter within the closed-cell version of the model, the bifurcation diagram of the closed-cell model is computed (Dupont et al., 2016), and then solutions of the full (open-cell) model are superimposed on the bifurcation diagram. This method of treating a slow-moving variable as a bifurcation parameter was pioneered by Rinzel (Rinzel, 1985; Rinzel and Lee, 1986) for the study of bursting in neuroendocrine cells and has proven useful for the analysis of Ca^2+^ models (Dupont et al., 2016).

In the present case, *δ* serves as a useful parameter for changing the speed of *c*_*t*_ relative to all the other variables. The value of *δ* used in many of our model simulations does not result in clear time-scale separation, but use of smaller values of *δ* does uncover more clearly the underlying relationships between the bifurcation structure of the closed-cell model and the open-cell solutions.

Although this approach does not constitute a rigorous timescale analysis, it serves as a valuable framework for comprehending the qualitative behavior of the model by treating it as a closed-cell system and utilizing its dynamics to make predictions about the qualitative behavior of the open-cell model.

#### 4.1.1 Thin spikes

**FIGURE 5:**
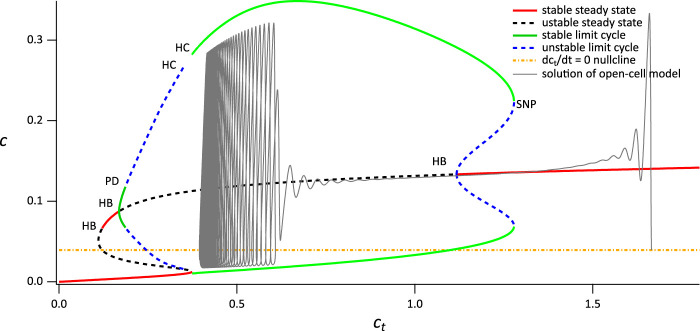
Partial bifurcation diagram of the closed-cell model using *c*_*t*_ as the bifurcation parameter. The grey curve is a solution of the open-cell model, and the yellow line is the $\frac{dc_{t}}{dt}$ nullcline for the full system. HB denotes a Hopf bifurcation, HC denotes a homoclinic bifurcation, PD denotes a period-doubling bifurcation, SNP denotes a saddle-node of periodics. Parameters are in Supplementary Appendix S6.2, Table 2, with *V*_PLC_ = 0.014 *μ*M s^−1^.

First consider the closed-cell model when *V*_PLC_ = 0.014 *μ*M s^−1^. The partial bifurcation diagram, using *c*_*t*_ as the bifurcation parameter, is shown in Figure 5. There are three features we note in particular:

1. The curve of steady states is folded, and exhibits bistability for a range of values of *c*_*t*_. This is not important for the thin spikes we discuss here, but will be critical later.
2. The upper branch of steady states contains two Hopf bifurcations, each of which serves as the origin of a branch of periodic orbits. The left-hand branch of periodic orbits goes unstable in a period-doubling bifurcation and ends in a homoclinic bifurcation. The right-hand branch also ends in a homoclinic bifurcation.
3. The *dc*_*t*_/*dt* = 0 nullcline intersects the branch of stable periodic orbits and also intersects one of the branches of unstable steady states.

The various homoclinic and period doubling bifurcations are of no further interest. Neither is the Hopf bifurcation at the lowest value of *c*_*t*_, nor its associated periodic branches.

The reason this bifurcation diagam is useful becomes apparent when we superimpose the solution of the open-cell model (shown in Figure 5 as a gray line). Before stimulation, the cell is sitting at a relatively high value of *c*_*t*_, just above 1.6 *μ*M. Upon stimulation, Ca^2+^ is released from the ER, and some of it is pumped out of the cell by the PMCA pumps, leading to a slow decrease in *c*_*t*_ (note that SOCE is absent). As *c*_*t*_ decreases slowly, the solution of the open-cell model approximately tracks the bifurcation diagram of the closed-cell model; the smaller *δ* is, the more accurate this tracking will be. For the value of *δ* we use here the solution of the open-cell model does not track the closed-cell bifurcation diagram exactly; however, the closed-cell model still provides a reasonable explanation for the behaviour of the open-cell model, and the qualitative agreement is clear.

Initially, the solution of the open-cell model collapses on to the upper stable branch of steady states of the closed-cell model. However, as *c*_*t*_ decreases, the upper branch of steady states becomes unstable in a Hopf bifurcation. Although the open-cell solution does not thereby immediately tend towards the stable periodic orbits (Baer et al., 1989) it does so eventually, and then tracks along the branch of stable periodic orbits. Eventually, *c*_*t*_ stops decreasing and the open-cell solution terminates in a stable periodic orbit that corresponds closely with the stable periodic orbit of the closed-cell model. Although it is possible to calculate exactly where the stable open-cell periodic solution lies, that is well beyond the scope of this paper.

The end result is a stable thin-spike solution of the open-cell model, as shown in Figure 3D. This thin-spike solution persists essentially unchanged if transport of Ca^2+^ across the cell membrane is blocked, i.e., if *c*_*t*_ is held fixed.

#### 4.1.2 Mixed responses

**FIGURE 6:**
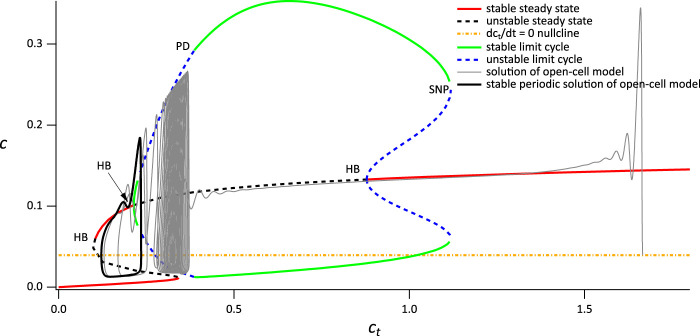
Partial bifurcation diagram of the closed-cell model using *c*_*t*_ as the bifurcation parameter. The grey curve is a solution of the open-cell model, and the yellow line is the $\frac{dc_{t}}{dt}$ nullcline for the open system. The rightmost Hopf Bifurcation is labeled HB; the leftmost Hopf bifurcation is obscured by the superimposed solution of the open-cell model. Parameters are in Supplementary Appendix S6.2, Table 2, with *V*_PLC_ =0.0145 *μ*M s^−1^.

In order to explain the combination of thin and broad spikes seen in Figure 3B, we increase *V*_PLC_ to 0.0145 (Figure 6). In this case, IP_3_ has a slightly higher concentration, the flux through the IP_3_ receptor is slightly higher, and thus *c*_*t*_ decreases slightly faster. Although the bifurcation diagram of the closed-cell model has essentially the same structure as the previous case (some minor details are different, such as the merging of the two branches of periodic orbits and consequent loss of the two homoclinic bifurcations, but these are of no interest for the present study) the open-cell solution now does not stabilise at a value of *c*_*t*_ that gives thin spikes. Instead, the solution crosses the leftmost Hopf bifurcation and returns to the stable upper branch of steady states, eventually falling to the lower branch. On this lower branch, *c*_*t*_ is increasing and so the solution moves to the right until it reaches the saddle node, whereupon it moves back to the upper branch and repeats the cycle. It is this cycling between the upper and lower branches that results in the broad spike.

Although the broad spike does not exactly follow the branches of closed-cell bifurcation diagram, this is partially due to the value of *δ* we use here, which does not result in a clear time-scale separation. Nevertheless, despite this lack of quantitative agreement, the basic outlines of the broad spike, alternating between the upper and lower branches of the closed-cell bifurcation diagram, is clear. Although preliminary computations suggest that, in the limit as *δ* → 0, the precise structure of the broad spike is more complicated than that suggested here, such an investigation is beyond the scope of this study.

It follows that the narrow and broad spikes observed in the time series in Figure 3A have quite different dynamical structures, a feature that was already highlighted in Cloete et al. (2020). In particular, as long as *c*_*t*_ is high enough, narrow spike oscillations will be observed in the closed-cell experiment as they do not require a consistent variation in *c*_*t*_, in contrast to the broad spikes, which will not be seen in a closed cell. The *c*_*t*_ nullcline (yellow line) intersects the critical manifold at an unstable region, explaining the persistent broad spike oscillation as the system has no stable steady solution.

#### 4.1.3 Broad spikes

**FIGURE 7:**
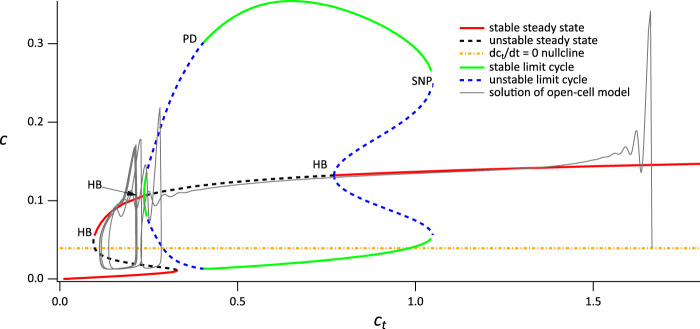
Partial bifurcation diagram of the closed-cell model, using *c*_*t*_ as the bifurcation parameter, with a superimposed solution from the open-cell model, computed with *V*_PLC_ = 0.015 *μ*M s^−1^. SN denotes a saddle node. Hopf bifurcations and branches of periodic orbits of no physiological relevance are omitted.

The outcome of the procedure using *V*_PLC_ = 0.015 is shown in Figure 7. Again, the bifurcation diagram of the closed-cell model has essentially the same structure. In the absence of agonist stimulation, the open-cell model has a resting *c*_*t*_ of around 1.6. Upon agonist stimulation, Ca^2+^ is released from the ER and is removed from the cell thus decreasing *c*_*t*_. The solution of the open system thus traces the upper stable branch of steady states in the closed system. As in the previous case, the solution passes through the Hopf bifurcation without immediately tending to the stable periodic orbit. However, in this case the solution moves past the lower Hopf bifurcation before it can reach the stable periodic orbit, resulting in the absence of any transient thin spikes.

When the solution reaches the lefthand saddle node (to be more precise, once the solution reaches a Hopf bifurcation located very close to the saddle node, but this detail is not relevant here), it falls down to the lower branch of stable steady states which lies below the *dc*_*t*_/*dt* = 0 nullcline. Thus, *c*_*t*_ begins to increase until it reaches the righthand saddle node, whereupon it switches back to the upper stable branch, and repeats the cycle. A more detailed explanation of the dynamics underlying the broad spike oscillation can be found in Cloete et al. (2020). Note that the fact that the *c*_*t*_ nullcline of the open system (yellow line) intersects the steady-state curve at an unstable steady state allows for the emergence of sustained broad spikes (Figure 3B) since there is no stable steady state of the open system.

#### 4.1.4 No steady-state oscillations

Finally, employing the parameter values from Figure 3C slightly alters the bifurcation diagram as well as the *c*_*t*_ nullcline of the open system, causing it to intersect the steady-state curve on a stable branch (computations not shown). As a result, we do not observe sustained oscillations in Figure 3C.

## 5 Discussion

The present study was undertaken to unravel the underlying mechanism governing the behavior of STIM1/2-KO HEK293 cells. Previous investigations (Emrich et al., 2021) provided single-dose experimental data of this cell type. Our subsequent data analysis demonstrated a wide range of cellular responses to stimulation, particularly unveiling the existence of various oscillation shapes within a single time series.

The heterogeneity observed in these oscillations prompted our research to concentrate on two primary purposes: firstly, to elucidate the origins of these distinct oscillation shapes within a singular time course and replicate them; and secondly, to reproduce the observed diversity of cellular behaviors.

To address these questions, we used a modeling approach, predicting the STIM1/2-KO cells’ behaviors to stem from a balanced regulation of IP_3_ concentration. This regulation was implemented through a balanced regulation of IP_3_ production and degradation. By varying the value of *V*_PLC_ we successfully reproduced the spectrum of cellular behaviors.

In accordance with Emrich et al. (2021), our model includes the sequential positive and negative regulation of IP_3_ receptors by cytosolic Ca^2+^. Our model also predicts that the thin spike oscillations are the result solely of Ca^2+^ regulation of the IP_3_ receptor (computations not shown), and can thus occur in a closed cell, while the broad spike oscillations rely on variations in both [IP_3_] and *c*_*t*_, and thus cannot occur in a closed cell.

These predictions can be tested in two ways, firstly by subjecting the cells to an IP_3_ pulse, using the experimental procedure outlined in Sneyd et al. (2006), and, secondly, by closing the cell (with, say, gadolinium at a sufficiently high concentration) at different times. If the cell is closed while thin spike oscillations are occurring, the model predicts that the spiking will continue essentially unaffected. However, if the cell is closed while broad oscillations are occurring, the model predicts that the oscillations will stop.

The underlying nature of dual mechanisms yielding narrow and broad Ca^2+^ spikes have been explored by Cloete et al. (2020). Their model closely resembles our model, differing only in two key assumptions: they considered IP_3_ degradation as a linear function of *p*, whereas our model supposes that IP_3_ degradation depends on cytosolic calcium. Additionally, their model did not eliminate SOCE. They conducted a bifurcation analysis adopting a methodology similar to that outlined in Sneyd et al. (2004) that we used ourselves. In their study, changing the *V*_PLC_ value unveiled two dynamic structures. The first (*V*_PLC_ = 0.6) replicated narrow spikes, while the second (*V*_PLC_ = 0.1) generated broad spikes.

Interestingly, the bifurcation diagram Cloete et al. identified for broad spikes shares a qualitatively similar dynamical structure with our finding for *V*_PLC_ = 0.015. However, the structure underpinning narrow spikes diverges from ours. Furthermore, none of the dynamical structures they identified allow for the presence of qualitatively distinct oscillations within a single time series. The structure we uncovered is then reaffirmed to hinge on a delicate balance between IP_3_ production and degradation that is regulated by cytosolic Ca^2+^.

This paper is only the latest contribution to a long line of papers studying the relationship between Ca^2+^ oscillations and IP_3_ oscillations. It has been known since the work of Tsien and Tsien (1990) and Cuthbertson and Chay (1991) that Ca^2+^ feedback on the production and degradation of IP_3_ is possibly an important mechanism underlying Ca^2+^ oscillations in a variety of cell types. This was explored theoretically by Dupont and Erneux (1997) and Kummer et al. (2000) (among others).

A Ca^2+^-dependent balance between IP_3_ production and degradation has been previously introduced in a model crafted by Politi et al. (2006). It is noteworthy, however, that their model is underpinned by assumptions markedly distinct from ours, with flux expressions derived from Li and Rinzel (1994), Lytton et al. (1992), and Camello et al. (1996). The sole similarity with the models we developed lies in the dynamic behavior of IP_3_. Also, their analysis concentrated on either positive or negative feedback, neglecting a detailed examination of a hybrid model.

The model here is essentially an extension of models based on the experimental work of Thomas (Bartlett et al., 2020; Cloete et al., 2020; 2021) and Trebak (Yoast et al., 2020; Emrich et al., 2021); which extended earlier models by including Ca^2+^ interactions with both PLC and PKC and studying how the underlying dynamical structures could give insight into physiological processes. None of these earlier studies investigated how oscillations with qualitatively different shapes could occur in a single time series.

Many of the parameters of the model are taken from the earlier models of Sneyd et al. (2017), Cloete et al. (2021) and Emrich et al. (2021). This raises two important questions; firstly, how accurate are these parameters for HEK293 cells, and, secondly, can one reasonably expect that Ca^2+^ oscillations in HEK293 cells and hepatocytes (the subject of Cloete et al. (2021)) result from the same mechanisms? The answer to the first question is that we do not know exactly; most of the parameters in the model have been measured in other cell types, but not directly in HEK293 cells. However, there is reason to believe that, say, IP_3_ receptors or SERCA pumps have similar fundamental properties in all cell types, and thus the parameters in those submodels can be transferred between cell types with some confidence. The second question has a similar answer. Although there is enormous diversity in the details, the fundamental fluxes and reactions that underlie Ca^2+^ oscillations appear to be broadly similar in many cell types, a fact which has led to the idea of the Ca^2+^ ‘toolkit’, a set of fluxes, common to most cell types, that can be combined in different ways to achieve a wide variety of outcomes (Bootman et al., 2001; Thul et al., 2008). On the other hand, our model cannot tell us that modulation of PLC is absolutely required in order to see such oscillations, or that additional mechanisms, such as modulation of PKC by Ca^2+^, are playing no role at all. It would be a mistake to draw such specific conclusions from the results shown here.

This emphasises the fact that a model such as this one should not be interpreted as a literal and quantitative description of Ca^2+^ dynamics in HEK293 cells. Rather, it is a realisation of the underlying dynamical structure—no matter how this structure is realized in practice—that will result in the correct qualitative behaviour seen in real cells. Such a dynamic approach was used by Sneyd et al. (2017) and has proven to be a useful way of guiding the kinds of experiments that can be done to help understand the underlying behaviours. For example, our analysis suggests that the presence or absence of Ca^2+^ fluxes across the plasma membrane play a crucial role in governing the shape of the oscillations. This, in turn, suggests that changes in *δ* (with, for example, gadolinium) would be an important way to probe the cell’s behaviour experimentally. Indeed, some of these experiments have already been done in HEK293 cells (Sneyd et al., 2004) and others are presented here.

Our study currently has an incomplete understanding of the IP_3_ balance, leaving us without a systematic method to reproduce qualitatively different oscillations along a single time series. Achieving a more general theoretical understanding of how this occurs would be valuable because it could then be applied to any model trying to reproduce a hybrid oscillation time series. For example, tracking all the bifurcations (including those we have not discussed in detail here) as functions of both *V*_PLC_ and *c*_*t*_ would be a useful start in characterizing the parameter regions associated with distinct behaviors, serving as an initial step toward unraveling the complexity of the IP_3_ balance mechanism. Such studies are left for future work.

Another aspect of this model that would benefit from additional investigation is the control of the width of the broad spikes. For instance, is it possible to generate broad spikes of arbitrary width simply by changing parameter values? Again, a detailed two-parameter bifurcation study would be the most obvious place to start in trying to answer this question.

Furthermore, it would be of great interest to broaden the scope of our study and include data from Emrich et al. (2021) that we overlook here, notably the data from cells in which only STIM1, or only STIM2, has been knocked out, as opposed to the double knockouts discussed here. We would apply a similar methodology to the one performed in this article, starting with a data analysis followed by a time series classification. We could then leverage the approach suggested by in Yoast et al. (2020), where they assigned a distinct *α*_1_ value (as found in the expression of *J*_in_) to each Orai KO combination, thereby modulating the influx of Ca^2+^ into the cell through SOCE. While this allowed them to replicate the primary features observed in the Orai knockout combination, it fell short of reproducing broad spikes (Yoast et al., 2020; Lee et al., 2024 under revision). By applying a similar approach to our newly developed model, we aspire to achieve a comprehensive reproduction of all cellular behaviors.

In conclusion, our investigation into the behavior of STIM1/2-KO HEK293 cells has provided novel insights into the complex dynamics of calcium oscillations. By employing a modeling approach, we uncovered a delicate balance between IP_3_ production and degradation regulated by cytosolic calcium. This study not only elucidated the diverse cellular responses observed but also paved the way for exploring qualitatively distinct oscillations within a single time series, offering new insights into the intricate realm of cellular signalling.

## Data Availability

The original contributions presented in the study are included in the article/Supplementary Material, further inquiries can be directed to the corresponding author.
