# Supplementary material for: Calcium oscillations in HEK293 cells lacking SOCE suggest the existence of a balanced regulation of IP3 production and degradation
Source: Front Syst Biol. 2024 Mar 15;4:1343006. doi: 10.3389/fsysb.2024.1343006 (PMC12341962; doi:10.3389/fsysb.2024.1343006)
Supplement: Supplementary file 1 [file DataSheet1.pdf]

6 APPENDIX

6.1 Data

	A	B	C	D
Set 1	28	33	45	14

	A	B	C	D
Set 2	22	30	39	29

Table 1. Classification of the Ca^{2+} responses for 2 sets of 120 STIM1/2-KO cells after stimulation by 10 µM of CCh during a single-dose experiment in open-cell conditions. Columns A, B, C, and D refer to the representative behavior illustrated in Fig. 1A, B, C, and D, respectively.

6.2 Model Equations

The model equations are

$$\frac{dc}{dt} = J_{\text{IPR}} - J_{\text{SERCA}} + \delta(J_{\text{in}} - J_{\text{PM}}), \tag{1}$$

$$\frac{dc_t}{dt} = \delta(J_{\text{in}} - J_{\text{PM}}), \tag{2}$$

$$\tau_h(c)\frac{dh}{dt} = h_\infty(c) - h, \tag{3}$$

$$\frac{dp}{dt} = \phi_L(c) - \phi_p(c)p, \tag{4}$$

where

$$c_e = \gamma(c_t - c), \tag{5}$$

$$\phi_L(c) = V_{\text{PLC}}\frac{0.78 + 0.5c^6}{K_n^6 + c^6}, \tag{6}$$

$$\phi_p(c) = A_2\frac{1 + 100c^{4.8}}{K_g^{4.8} + c^{4.8}}, \tag{7}$$

$$J_{\text{in}} = \alpha_0, \tag{8}$$

$$J_{\text{PM}} = V_{\text{PM}}\frac{c^2}{K_{\text{PM}}^2 + c^2}, \tag{9}$$

$$J_{\text{SERCA}} = V_{\text{SERCA}}\frac{c^2 - \hat{K}c_e^2}{c^2 + K_{\text{SERCA}}^2}, \tag{10}$$

$$J_{\text{IPR}} = k_f P_0(c_e - c). \tag{11}$$

The model of the IP_3 receptor, taken directly from Sneyd et al. (2017) is

$$P_0 = \frac{\beta}{\beta + 0.4(\beta + \alpha)}, \tag{12}$$

$$\alpha = A(p)(1 - m_\alpha(c)h_\alpha(c)), \tag{13}$$

$$\beta = B(p)m_\beta(c)h, \tag{14}$$

$$m_\alpha(c) = m_\beta(c) = \frac{c^4}{K_c^4 + c^4}, \tag{15}$$

$$h_\alpha(c) = h_\infty(c) = \frac{K_h^4}{K_h^4 + c^4}, \tag{16}$$

$$1 - A(p) = B(p) = \frac{p^2}{K_p^2 + p^2}, \tag{17}$$

$$\tau_h = \tau_{\max}\frac{K_\tau^4}{K_\tau^4 + c^4}. \tag{18}$$

Note that both the production and degradation of IP_3 are assumed to be increasing functions of $[Ca^{2+}]$. By appropriate parameter selection, the steady-state value of p can thus be made a biphasic function of c. Also, in the case of STIM1/2-KO cells, $J_{\text{in}} = \alpha_0$ is assumed to be constant, as the flux coming from SOCE is abrogated (Dupont et al., 2016).

The model parameters are given in Table 2.

Parameter	Value	Parameter	Value
α_0	0.007 (0.0006)	δ	0.176
V_{PM}	0.186 (0.12)	K_{PM}	0.2 (0.14)
V_{SERCA}	0.36	K_{SERCA}	0.2
$\hat{K}$	0.00001957	$\tau_{\max}$	75
k_f	1.2	γ	55
K_c	0.14	K_h	0.08
K_τ	0.1	K_n	0.214
V_{PLC}	0.014	K_p	0.2 μM
K_g	0.275	A_2	0.104

Table 2. Model parameters. The values in braces are used to generate the plots in Fig. 3C. All concentrations are in μM and time is in seconds. These parameters are taken from previous models (Sneyd et al., 2017; Cloete et al., 2021; Emrich et al., 2021), slightly adjusted to obtain qualitatively accurate oscillation periods.
